# Existence of hepatitis B virus surface protein mutations and other variants: demand for hepatitis B infection control in Cambodia

**DOI:** 10.1186/s12879-020-05025-3

**Published:** 2020-04-25

**Authors:** Ko Ko, Kazuaki Takahashi, Shintaro Nagashima, Chikako Yamamoto, Vichit Ork, Aya Sugiyama, Tomoyuki Akita, Masayuki Ohisa, Channarena Chuon, Md. Shafiqul Hossain, Bunsoth Mao, Junko Tanaka

**Affiliations:** 1 Department of Epidemiology, Infectious Disease Control and Prevention, Hiroshima University Graduate School of Biomedical and Health Sciences, Japan; 1-2-3 Kasumi, Minami-ku, Hiroshima, 734-8551 Japan; 2 National Immunization Programme, Ministry of Health, 151-152 Kampuchea Krom Avenue, Phonm Penh, Cambodia; 3 Expanded Programme on Immunization, World Health Organization Country Office, Cambodia; 61-64, Preah Norodom Blvd (St 306), Sangkat Boeung Keng Kang I, Khan Chamkamom, Phnom Penh, Cambodia; 4 University of Health Sciences, 73 Monivong Boulevard, Phnom Penh, Cambodia

**Keywords:** Genotype, Full genome sequence, Nationwide, Mutation, Hepatitis B virus, Dried blood spot, Cambodia

## Abstract

**Background:**

This study aimed to detect Hepatitis B virus (HBV) genome sequences and their variants as of nationwide scale using dried blood spot (DBS) samples and to provide up-to-date reference data for infection control and surveillance in Cambodia.

**Method:**

Among 2518 children age 5–7 years and their 2023 mothers participated in 2017 Cambodia nationwide sero-survey on hepatitis B surface antigen (HBsAg) prevalence using multistage random sampling strategy, 95 mothers and 13 children positive to HBsAg were included in this study. HBV DNA was extracted from DBS, then performed polymerase chain reaction. HBV genotypes and potential variants were examined by partial and full length genomic analysis.

**Results:**

HBsAg positive rate was 4.7% (95/2023) in mothers and 0.52% (13/2518) in their children. Genotype C (80.49%) was abundantly found throughout the whole Cambodia whilst genotype B (19.51%) was exclusively found in regions bordering Vietnam. S gene mutants of HBV were found in 24.29% of mothers and 16.67% of children with HBV DNA positive sera. Full-length genome analysis revealed the homology of 99.62–100% in each mother-child pair. Genotype B was clarified to recombinant genotype B4/C2 and B2/C2. Double (48.39%) and combination mutation (32.26%) were observed in core promoter region of HBV C1 strains.

**Conclusions:**

This study showed the capable of DBS for large-scale molecular epidemiological study of HBV in resource limited countries. Full-genome sequences yield the better understanding of sub-genotypes, their variants and the degree of homology between strains isolated from mother-child pairs calls for effective strategies on prevention, control and surveillance of mother-to-child HBV transmission in Cambodia.

## Background

Viral hepatitis infection including hepatitis B virus (HBV) is still challenging as the public health concern, having global prevalence of 3.5% and 1.34 million deaths in 2015 [1]. The prevalence of HBV infection might differ in each World Health Organization (WHO) region [2] and the high prevalence of 6.1 and 6.2% were found in Africa and Western Pacific region respectively [1].

Although the gross decrement of HBV prevalence was reported in the developed countries after discovery of effective hepatitis B vaccine (HepB) since 1981 [3], the prevalence is still high in developing countries. Mother-to-child transmission (MTCT), also known as vertical transmission, still ranks as the main route of HBV transmission in intermediate and high endemic countries. Cambodia, one of the developing countries in WHO Western Pacific Region, has been reported high hepatitis B surface antigen (HBsAg) prevalence ranging from 7.7 to 13% [4, 5]. In 2005, Cambodia started phasing-in HepB vaccine to National Immunization programme (NIP) and the coverage was achieved over 90% since 2008 [6]. After introduction of HepB vaccine in the whole Cambodia, HBsAg prevalence among ≤5 years old children markedly reduced to 3.5% in 2006 [7] and then dropped to 0.33–3.45% in three provinces of Cambodia in 2011 [5]. Recent nationwide study on HBsAg prevalence among mother-child pairs in 2017 revealed the positive rate of 0.56% among children and 4.39% among their mothers [8]. Very low HBsAg positive rate in children with its reciprocal high positive rate in mothers indicates the needs for further study on HBV in Cambodia. Moreover, the clinical outcomes of chronic HBV infection rely on HBV genotypes and sub-genotype as viral factor. Understanding HBV genotypes and sub-genotypes can predict not only liver disease progression but also the response to antiviral treatment [9]. Although there were only a few reports about HBV genotype distribution in Cambodia [4, 10], the nationwide distribution pattern of HBV genotypes was still unknown.

Additionally, the widespread use of HepB vaccine in combating HBV infection potentially threatens the emergence of mutant strains at hepatitis B surface gene. The mutation in S gene causes the amino acid substitution either single or multiple mutations in HBsAg especially *a* determinant region between amino acid 120 and 147 and mutation in this region reduces the sensitivity to diagnostic test, failure of response to both HepB vaccine and HBIG [11]. It is later denoted as vaccine escapes mutation and is abundantly occurred in those children who had received plasma-derived vaccines (0.3%) rather than recombinant vaccines (0.06%) [12]. The emergence of vaccine escapes mutants threatens the efficacy of HepB vaccine among infants and now raising as the public health concern in elimination pathway of HBV. Although Cambodia has a long track of using HepB vaccine over a decade, there is no study on S gene mutation of HBV meanwhile.

Therefore, this study aimed to detect HBV genome sequences and their potential mutant strains specifically mutation at S gene of HBV as of nationwide scale using dried blood spot (DBS) samples and then to provide the up-to-date reference data for consideration of prevention, control and surveillance of HBV infection in Cambodia.

## Methods

### Subjects of the study

This was the nationwide sero-epidemiological study on HBsAg prevalence among 5–7 years old children and their mothers from 25 provinces of the whole Cambodia in 2017 using the multistage stratified random sampling strategy. Its study designs was introduced previously [8] and results of HBV prevalence from this study had been accepted by WHO Western Pacific Regional Office. Dried blood spot (DBS) using HemaSpot™ (Spot on Science Inc., Austin, USA) samples were collected from 2520 children and their 2028 mothers but two children DBS samples and five mothers’ DBS samples were excluded for their insufficient amount of blood for measurement. Therefore, a total of 4541 DBS samples (2518 children and 2023 mothers) were tested for HBsAg (LumipulseII® HBsAg, Fujirebio, Japan with reported sensitivity of 100% and specificity of 99.7% [13]) by chemiluminescent enzyme immunoassay (CLEIA) using Lumipulse G1200 (Fujirebio Inc., Japan) with cut-off value of 1.0. The reported sensitivity and specificity of HBV DNA using DBS was 95% (95% CI: 83–99) and 99% (95% CI: 53–100), respectively [14, 15]. The vaccination history was taken from yellow book (the vaccination records) provided by Ministry of Health of Cambodia. The recall memory on vaccination status was also taken from the parents or guardians of those children whose yellow books were not present.

### Nucleic acid extraction

HemaSpot**™** contains 8 fins of filter papers and the nucleic acid was directly extracted from one fin of HBsAg positive DBS samples using SMITEST EX-R&D (Medical and Biological Laboratories co., LTD, MA, USA) strictly following the manufacturer’s instruction. The final pellets highly concentrated with nucleic acid were then suspended in 50 μl of distilled water and then performed the polymerase chain reaction (PCR).

### Partial and full-length genomes sequencing

For full-length genome sequences, the same primers as of the previously described method were used in this study [16, 17]. The amplification was carried out by nested polymerase chain reaction (Nested-PCR) using Prime STAR^®^GXL polymerase (Takara Bio Inc., Shiga, Japan) and the primer set A (WA-L and WA-R and inner primers WA-L2 and WA-R2) [16]. For the missing portion of the circular HBV DNA, the extracted DNA was assigned again for the nested PCR using Prime STAR^®^GXL polymerase (Takara Bio Inc., Shiga, Japan) and the primer set B (S1, S2, AS1, and AS2). The obtained PCR product was directly sequenced using a 3730xl DNA sequencer (Thermo Fisher Scientific K.K., Kanagawa, Japan) and the BigDye Terminator v3.1 Cycle Sequencing Kit (Applied Biosystems, Foster City, CA, USA).

The samples which were not detected by WA primer set, were then attempted for s-region fragment (partial genome sequence) using the primer set #S1–1 and #S1–2 and the inner primers #S2–1 and #S2–2 [18, 19]. The obtained PCR products were directly sequenced as the same way mentioned in full length sequences.

### Molecular evolutionary analysis

The sequence data were analyzed by GENTYX-MAC Version 18 software (Genetyx Corporation, Tokyo, Japan). Genotypes B1-B9 and C1-C16 obtained from GenBank were assigned as reference standard strains for sequencing. The further analysis of genotype C1 was done by the neighbor-joining method [20] and then the evolutionary analysis of Texa was employed in MEGA7 [21].

### Detection of HBV genome recombination

The recombination of circular HBV DNA was detected using the SimPlot program and boost scanning analysis [22] with jumping profile Hidden Markov Model (jpHMM) for recombination detection in circular genomes [23]. 11 HBV genotype B strains from this study were employed for the determination of HBV genome recombination and visualized in a circular form using the software package Circos [24].

### Statistical analysis

The statistical analysis was performed using JMP version 10 (SAS Institute Inc., Cary, NC). The χ^2^ and Fisher’s exact test were used appropriately to compare between groups. The statistical significance was set at *p* < 0.05.

## Results

### Study participants

Of 2023 mothers and 2518 children aged 5–7 years, HBsAg positive rate was 4.7% (95/2023) in mothers and 0.52% (13/2518) in their children and all HBsAg positive samples were included in this study. The mean age of mothers was 32.36 ± 6.01 years. 69.2% of children were 5 years old and 30.8% were 6 years old. Among 95 HBsAg positive mothers, nine of their children were positive for HBsAg giving MTCT rate of 9.5% (9/95). The detail of background demography were already discussed by Vichit et al. [8]. In this study, we present the outcomes from genome sequences analysis.

### Nucleic acid extraction and HBV genomes amplification

HBV DNA amplified by WA region primer set was detected in 52 samples (41 mothers and 11 children) from which the full genome sequences having 3 k base pairs (3kbp) could perform in 78.1% (32/41) of mothers and 90.9% (10/11) of children. After another trial of amplification to those samples undetected by WA primers, the partial sequencing using s-region primers was achieved in the HBV DNA positive samples of 29 mothers and 1 child. Therefore, HBV DNA was extracted from 73.7% (70/95) of mothers and 92.3% (12/13) of children who were positive for HBsAg and all these 82 samples were able to classify HBV genotypes in Cambodia. (Table 1).

**Table 1:**
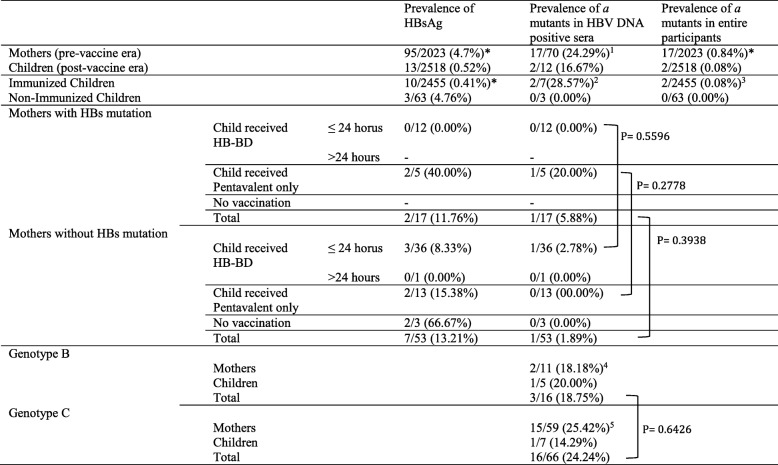
Prevalence of HBsAg and *a* determinant Variants in Cambodia

*: *p* < 0.001, 1: *p* = 0.5657, 2: *p* = 0.21, 3: *p* = 0.8223, 4: *p* = 0.9333, 5: *p* = 0.5191

### Nationwide HBV genotype distribution and phylogenetic tree

**Fig. 1:**
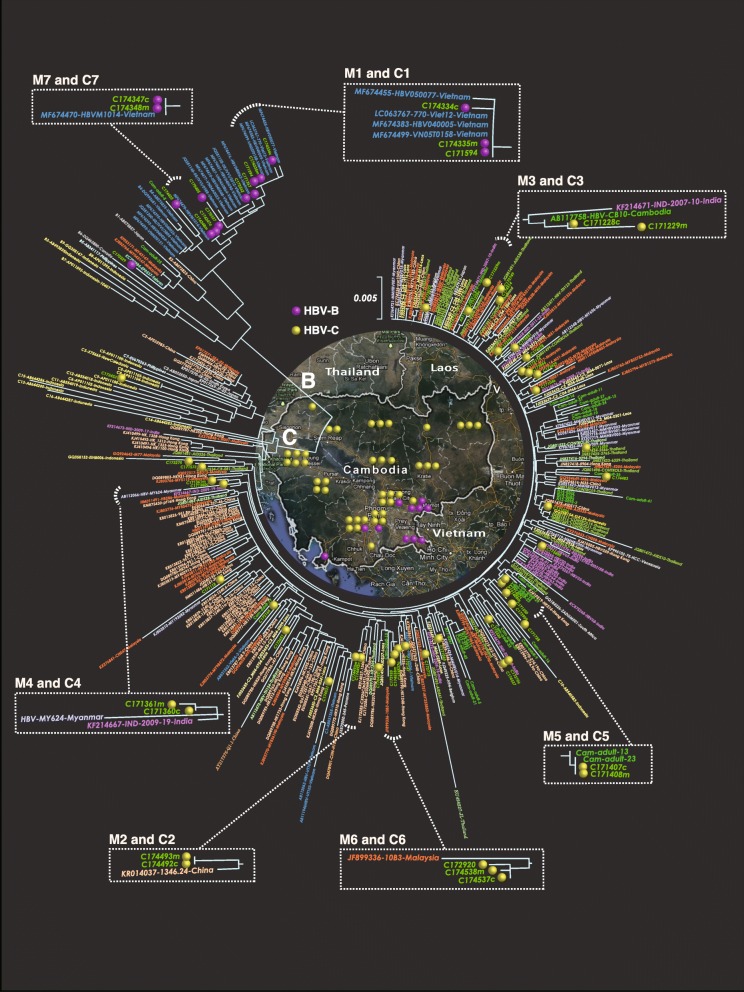
Countrywide genotype distribution of detected HBV strain from children and mothers in Cambodia. This figure shows the genotype distribution of the detected HBV strains among 5–7 years old children and their mothers in each province of Cambodia. HBV genotype B was represented by purple dot whereas HBV genotype C was indicated by yellow dot

HBV genotype was determined by the s-region of each detected strain using the neighbor-joining method. HBV genotype C was abundantly found in 84.3% (59/70) of mothers and 58.3% (7/12) of children. HBV genotype B was found in 15.7% of mothers (11/70) and 36.3% of children (5/12). As the phylogenetic tree was constructed by the strains having 823 base pairs from nt111-nt933, 53 out of 82 HBV DNA positive samples could assign. Almost all HBV genotype C were sub-grouped to C1 and were gathered in the same cluster of China, Hong Kong, Thailand, Laos, Malaysia, Myanmar and India except one (C173433) which is sub-genotype C 8 and is much closed to Indonesian strain (Fig. 1). Only a small portion of HBV genotype B was circulated in Cambodia and is in the same cluster to Vietnam in phylogenetic tree except one (C170329) which is adjacent to Taiwanese strain (Fig. 1).

### Homology of genome sequences in 7 mother-child pairs

Of 9 HBsAg positive mother-child pairs, 2 pairs were excluded for mothers’ refusal to participate. Among them, only one pair could amplify 2630 bp. The rate of base sequences match (homology) in six mother-child pairs ranged from 99.62 to 100%. The analysis of 2630 bp (nt1929-nt1343) detected from one mother-child pair (C171408m and C171407c) showed a 99.96% homology in their nucleotide sequence. (Table 2).

**Table 2:** Homology of the detected full length HBV strains isolated from 6 mother-child pairs Identity (%)

Isolate Name	C171228c	C171229m	C171360c	C171361m	C174334c	C174335m	C174347c	C174348m	C174492c	C174493m	C174537c
gC1 C171229m	99.62										
gC1 C171360c	98.53	98.25									
gC1 C171361m	98.35	98.25	99.78								
gB4/C C174334c	91.91	92.02	91.84	91.78							
gB4/C C174335m	91.94	92.05	91.87	91.81	99.9						
gB4/C C174347c	91.75	91.93	91.69	91.63	98.25	98.28					
gB4/C C174348m	91.78	91.96	91.72	91.66	98.28	98.32	99.96				
gC1 C174492c	98.38	98.25	98.22	98.25	92.06	92.09	92.03	92.6			
gC1 C174493m	98.38	98.25	98.22	98.25	92.06	92.09	92.03	92.6	100		
gC1 C174537c	98.5	98.16	98.56	98.41	91.81	91.85	91.75	91.81	98.28	98.28	
gC1 C174538m	98.47	98.13	98.53	98.44	91.78	91.81	91.72	91.75	98.25	98.25	99.87

The base sequence of the detected HBV strains from 6 mother-child pairs has homology from 99.62–100%

Analysis of up to 2630 bp (nt1929-nt1343) detected from the mother (C171408m) and child (C171407c) showed a 99.96% homology in the nucleotide sequence

gC1: HBV sub-genotype C1, gB4/C: recombinant HBV genotype B4/C, The isolate ID ends in “m” represents for mother and that ends in “c” represents for child

### Occurrence of S gene mutant strains of HBV in Cambodia

**Fig. 2:**
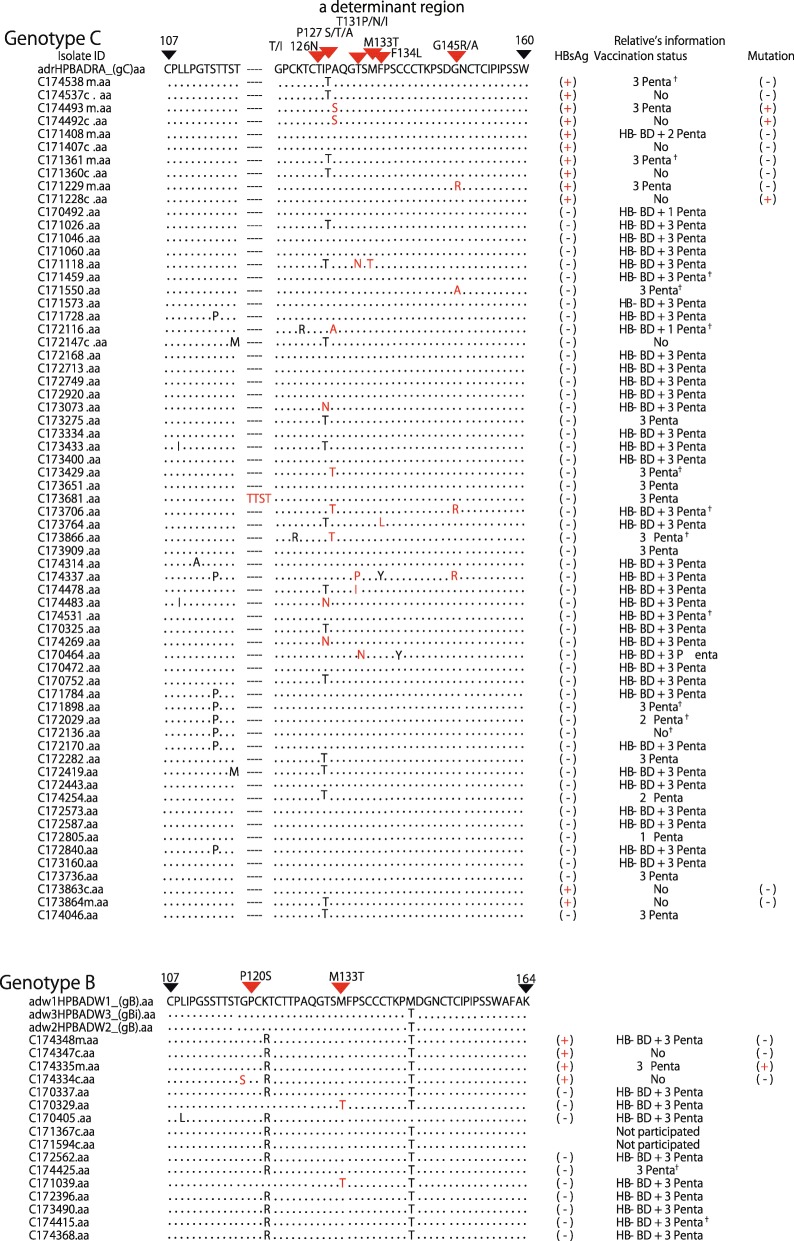
S gene mutation of HBV within a determinant region and its counterpart HBsAg and vaccination status. This figure shows the existence of S gene mutation of hepatitis B virus found within α determinant region from nt120-nt147 of either mother or child with additional HBsAg and vaccination status counterpart relatives. The relative refers to: if the isolate sample is mother, the relative information is for her child and vice versa. The isolate ID ended with “c” represents to “child” and “m” to “mother”. “*a* determinant region” will confine to “nt120-nt147”. †: The vaccination history was received by recall memory for those children whose vaccination card was absent at the time of survey

The determination of mutation was confined to the area specific for “*a* determinant region of hepatitis B surface antigen (nt121- nt149) [25]. S gene mutant strains of HBV were detected in 17 mothers and 2 children and were recognized at P120S, T/I126N, P127S/T/A, T131I/N, M133T, F134 and G145R/A. In children, one for each aa120 (P120S) and aa127 (P127S) were found (Table 3). But in mothers, the predominant mutation was found at aa127 (P127S/T/A: 5/17) and aa131 (T131I/N/P: 4/17) and aa145 (G145R/A: 4/17), aa126 (T/I126N: 3/17) and aa133 (M133T: 3/17), aa134 (F134L: 1/17) (Fig. 2). The overall S gene mutation rate among HBV DNA positive sera was 24.3% (17/70) in mothers and 16.7% (2/12) in children (*p* = 0.5657). The S gene mutation rate of HBV by the entire participants was 0.8% (17/2023) in mothers and 0.08% (2/2518) in children (*p* < 0.001). Among 17 mothers infected with S gene mutant of HBV, two children (11.7%) were found to be HBsAg positive. Among 53 mothers infected with wild type HBV, 7 children (13.2%) were positive to HBsAg (Table 4, Fig. 2).

**Table 3:** HBV Genotype, Mutation and Vaccination status of 13 HBsAg positive children and their mothers

		Children					Mother				
No.	Province	Sample ID	HBV DNA	Genotype	Mutation	Vaccination status	Sample ID	HBsAg	HBV DNA	Genotype	Mutation
1.	Pursat	c171228c	+	C	–	3 Penta	c171229m	+	+	C	**G145R**
2.	Kampong Thom	c174492c	+	C	**P127S**	3 Penta	c174493m	+	+	C	**P127S**
3.	Tbong Khmum	c174334c	+	B/C	**P120S**	HB-BD + 3 Penta	c174335m	+	+	B/C	–
4.	Preah Sihanouk	c174425c	+	B/C	**–**	HB-BD + 3 Penta	c174426m	–	–	–	–
5.	Phnom Penh	c171360c	+	C	–	No vaccination	c171361m	+	+	C	–
6.	Ratanak Kiri	c172147c	+	C	–	No vaccination	c172148m	–	–	–	–
7.	Stung Treng	c173863c	+	C	–	No vaccination	c173864m	+	+	C	–
8.	Preah Vihear	c174537c	+	C	–	No vaccination	c174538m	+	+	C	–
9.	Kampong Speu	c171407c	+	C	–	HB-BD + 2 Penta	c171408m	+	+	C	–
10.	Tbong Khmum	c174347c	+	B/C	–	HB-BD + 3 Penta	c174348m	+	+	B/C	–
11.	Ratanak Kiri	c172153c	ND			No vaccination	c172154m	+	+	C	–
12.	Svay Rieng	c171594c	+	B/C	–	HB-BD + 3 Penta	Not participated				
13.	Phnom Penh	c171367c	+	B/C	–	1 Penta	Not participated				

ND: not detected, B: HBV genotype B, C: HBV genotype C, HB-BD: birth dose hepatitis B vaccine, Penta: pentavalent vaccine including hepatitis B vaccine

**Table 4:** “*a*” determinant Variants of Mother and their corresponding child’s HBV and Immunization status

Mother					Child						Notes
Isolate ID	Age class	HBsAg/ Anti-HBs	Genotype/ sub-genotype	Mutant	Isolate ID	Age	HBsAg/ Anti-HBs	Genotype/ sub-genotype	Mutant	HB-BD/ Penta	Reported Function
C174493m	40–49	+/−	C1	P127S	C174492c	5	+/−	C1	P127S	−/3	Altered hydrophilicity, electrical charge and acidity of the loop
C171229m	30–39	+/−	C1	G145R	C171228c	5	+/−	C1	–	−/3	***Vaccine Escapes Mutant***
C170329	30–39	+/−	B2/C2	M133T	C170328	6	−/+	–	–	+/3	Rescue of virion secretion
C171039	30–39	+/−	^†^B	M133T	C171038	6	−/+	–	–	+/3	Rescue of virion secretion
C171118	20–29	+/−	^†^C	T131N, M133T	C171117	5	−/−	–	–	+/3	Rescue of virion secretion, Rescue of virion secretion
C171550	20–29	+/−	C1	G145A	C171549	6	−/+	–	–	−/3^‡^	***Vaccine Escapes Mutant***
C172116	20–29	+/−	C1	P127A	C172115	6	−/−	–	–	+/1^‡^	Unclear
C173073	30–39	+/−	^†^C	I126N	C173072	5	−/−	–	–	+/3	***Vaccine Escapes Mutant***
C173429	20–29	+/−	^†^C	P127T	C173428	6	−/+	–	–	−/3^‡^	Unclear
C173706	30–39	+/−	^†^C	P127T, G145R	C17305	5	−/−	–	–	+/3^‡^	Unclear, ***Vaccine Escapes Mutant***
C173764	20–29	+/−	C1	F134L	C173763	5	−/+	–	–	+/3	Lower reactivity in HBsAg assay
C173866	20–29	+/−	C1	P127T	C173865	6	−/+	–	–	NA/3^‡^	Unclear
C174337	30–39	+/+	C1	T131P, G145R	C174336	6	−/+	–	–	+/3	Unclear, ***Vaccine Escapes Mutant***
C174478	20–29	+/+	C1	T131I	C174477	6	−/+	–	–	+/3	Altered hydrophilicity, electrical charge and acidity of the loop
C174483	20–29	+/−	^†^C	I126N	C174482	5	−/−	–	–	+/2	***Vaccine Escapes Mutant***
C174269	30–39	+/+	^†^C	I126N	C174268	5	−/+	–	–	+/3	***Vaccine Escapes Mutant***
C170464	30–39	+/−	^†^C	T131N	C170463	5	−/−	–	–	+/3	Rescue of virion secretion

HB-BD: Birth dose Hepatitis B vaccine, Penta: Pentavalent vaccine including Hepatitis B Vaccine, HBsAg: Hepatitis B surface antigen, Anti-HBs: Hepatitis B surface antibody, M: Male, F: Female, NA: No information about immunization status, †: Full-length genome cannot be done, ‡: Immunization status was taken on recall memory of parents/guardians because no yellow book in hand during survey

HBsAg prevalence and S gene mutation rate of HBV among immunized children were 0.4 and 0.08% and that among non-immunized children were 4.8 and 0% respectively. The *a* determinant mutation rate among children infected from mother with mutant variant is higher than those infected from mother with wild type (5.9% Vs 1.9%). If the child received hepatitis B vaccination birth-dose (HepB-BD) within 24 h after birth, the infection rate among children with mutant variants is (2% Vs 4.5%). By each genotype, the mutation rate in genotype C was 24.2% (16/66) and that of genotype B was 18.8% (3/16).

### Characteristics of S gene mutant strains of HBV found among 13 mother-child pairs

After excluding children with undetectable HBV DNA (*n* = 1), whose mothers’ HBsAg negative (*n* = 2) and whose mothers refused to participate (n = 2), 8 mother-child pairs were then analyzed for S gene mutation of HBV. One mother-child pair has mutation at nt127 (P127S) in both mother and her child, one mother-child pair had mutation at nt120 (P120S) only in child and another one pair has mutation at nt145 (G145R) only in mother. Seven out of 13 children had completed at least 2 doses of pentavalent vaccine with or without HepB-BD. (Fig. 2).

### Double and combination mutant strains among children and their mothers in Cambodia

The double mutation at A1762T/G1764A was found only in HBV genotype C1 strains (12 mothers and 3 children) with the mutation rate of 48.39%. The combination mutation at C1653T and A1762T/G1764A or T1753C and A1762T/G1764A was also only found in HBV genotype C1 strains (10 mothers) with the mutation rate of 32.26%. (Table 5).

**Table 5:** Demography and Characteristic of 42Cambodian strains with mutation in Enhancer II and Core Promoter region

No.	Isolate Name	Age class	Residence	Genotype	Length (bp)	PreS Deletion	Enhancer II and Core Promoter		
							1653	1753	1762/1764
1.	C171361m	20–29	PP	C1	3215		(+)		
2.	C171360c	0–9	PP	C1	3215				(+)
3.	C174538m	20–29	PV	C1	3215		(+)		(+)
4.	C174537c	0–9	PV	C1	3215				(+)
5.	C171229m	30–39	PS	C1	3170	(+)			
6.	C171228c	0–9	PS	C1	3215				(+)
7.	C174493m	40–49	KT	C1	3215				
8.	C174492c	0–9	KT	C1	3215				
9.	C174335m	30–39	TK	B4/C2	3215				
10.	C174334c	0–9	TK	B4/C2	3215				
11.	C174348m	30–39	TK	B4/C2	3215				
12.	C174347c	0–9	TK	B4/C2	3215				
13.	C171459	30–39	PP	C1	3194			(+)	(+)
14.	C171060	20–29	KD	C1	3215		(+)		(+)
15.	C172116	20–29	KC	C1	3215				
16.	C172168	20–29	KC	C1	3191	(+)			
17.	C173909	20–29	KC	C1	3215			(+)	(+)
18.	C170492	30–39	KS	C1	3215				
19.	C171046	30–39	KS	C1	3215				
20.	C172562	20–29	KS	B4/C2	3215				
21.	C170405	40–49	KCh	B4/C2	3215			(+)	
22.	C171573	40–49	KCh	C1	3215			(+)	(+)
23.	C173681	30–39	KCh	C1	3227				
24.	C174314	30–39	KCh	C1	2961	(+)			
25.	C173866	20–29	ST	C1	3215				(+)
26.	C171550	20–29	SR	C1	3158	(+)			
27.	C174478	20–29	OM	C1	3215				
28.	C173275	30–39	TA	C1	3161	(+)		(+)	(+)
29.	C171728	20–29	PS	C1	3196				
30.	C172920	30–39	KT	C1	3215				
31.	C173764	20–29	KT	C1	3173	(+)	(+)		(+)
32.	C173334	30–39	BT	C1	3215				(+)
33.	C172749	30–39	BT	C1	3215			(+)	(+)
34.	C171026	30–39	BT	C1	3215				(+)
35.	C173400	40–49	BT	C1	3215				
36.	C170329	30–39	SRi	B2/C2	3215				
37.	C174337	30–39	TK	C1	3194	(+)	(+)		(+)
38.	C170337	20–29	SRi	B4/C2	3215				
39.	C171367c	0–9	PP	B4/C2	3239				
40.	C172147c	0–9	RK	C1	3215				
41.	C174425c	0–9	PSi	B4/C2	3215				
42.	C171594c	0–9	SRi	B4/C2	3215				

*PP* Phnom Penh, *PV* Preah Vihear, *PS*Pursat, *KT* Kampong Thom, *TK* Tbong Khmum, *KD* Kandal, *KC* Kratie, *KS* Kampong Speu, *KCh* Kampong Cham, *ST* Stung Treng, *SR* Siem Reap, *Sri* Svay Rieng, *OM* Otdar Meanchey, *TA* Takeo, *BT* Battambang, *RK* Ratanak Kiri, *PSi* Preah Sihanouk, “M”: Male, “F”: Female, “C1”: hepatitis B virus subgenotype C1, “B/C”: hepatitis B virus recombinant B/C, “(+)” indicates mutation occur in the core promoter, PreC and Core regions

### Full-length genome sequences and evolutionary analysis of Texa

**Fig. 3:**
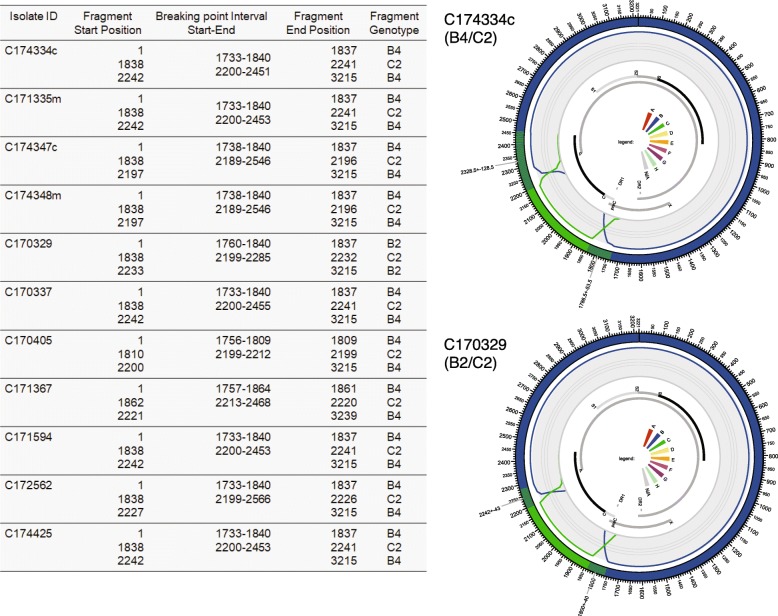
Structural model for Recombinant HBV genomes of this study. The figure shows the recombinant HBV genomes found in this study. The starting points and breaking points are shown in left side table. 11 HBV genotype B strains were enrolled for the detection of recombinant HBV genomes using jpHMM software. The structural configurations of the detected HBV recombinant genomes are shown in right figures; the upper one for recombinant B4/C2 and the lower one for recombinant B2/C2

We could do the full-genome sequences in 42 samples (32 mothers and 10 children) with the nucleotide length from 3161 to 3239 base pairs amongst which 31 strains were belong to genotype C and the rest (11 strains) were genotype B. All HBV genotype C belongs to sub-genotype C1 which were assumed to be originated from Indonesia, Thailand, India, China and Vietnam. For HBV genotype B, almost all detected strains (*n* = 10) are found to be recombinant genotype B4/C2. Only one strain (C170329) showed recombinant B2/C2. All these recombinant B/C strains build up with circular DNA mixing up of sequences resembling genotype B and a short portion of genotype C in core region (Fig. 3) with various breaking points for recombination. By mean of evolutionary relationship of Texa, all recombinant genotype B4/C2 strains are near to Vietnamese strains but B2/C2 is very near to Taiwanese strain.

## Discussion

This study is the first report to present HBV DNA positive rate, its amplification rate, genotype distribution and existence of potential HBV variants among the strains isolated from mother-child pairs in Cambodia as of its nationwide scale.

The overall HBV DNA positive rate in children was 0.48% which definitely reflects the well-established vaccination program in Cambodia. But, MTCT rate was 9.47% (9/95) which is higher than the previously reported rate among vaccinated Asian (2–3%) [26]. The homology between HBV strains isolated from these mother-child pairs was 99.62–100% which strongly indicated that the transmission was vertical.

The genome sequences revealed the genotype distribution pattern of HBV in the whole Cambodia. HBV genotype C was abundantly found in almost all provinces of Cambodia except Kandal, Kampong Cham, Tbong Khmum and Svay Rieng provinces where HBV genotype B was predominant. Some studies also reported that genotype C is the predominant genotype in Cambodia and then followed by genotype B [10, 27]. Cambodia is bordered by Thailand to the northwest, Laos to the northeast, and Vietnam to the east and the Gulf of Thailand to the southwest. The genotype distribution pattern is linked to the neighboring countries of Cambodia. In our study, HBV genotype B was exclusively circulated in Svay Rieng, Kampot and Tbong Khmum provinces; the border regions to the Vietnam where HBV genotype B is predominant (72.6%); particularly sub-genotype B4 (87.8%) [28]. HBV genotype C was abundantly found in the Stung Treng, Ratanak Kiri and Preah Vihear provinces, the northeast part of Cambodia and border region to Laos, where HBV genotype C (55.4%) is also predominant [29]. Meanwhile, in Otdar Meanchey, Pursat and Battambang provinces; the west and northwest regions of Cambodia bordering to Thailand, HBV genotype C was exclusively found where 73 to 87.5% of the detected HBV strains were genotype C [30, 31]. In fact, HBV genotypes B and C are the most prevalent types in Asia and the genotype C has more pathogenicity in compared with genotype B [32]. By this study, it is supposed to have the historical relation of HBV genotype between Cambodia and its neighboring countries. Therefore, this nationwide genotype distribution pattern raises two important issues for the infection control of HBV in Cambodia. Firstly, the health sector should be aware of geographic variation of HBV genotypes and its historical relation among neighboring countries and should consider for implementation of effective HBV preventive strategies among migrants from both sides. Secondly, the reported predominant genotypes and sub-genotypes of our study can be the clue for better understanding of viral factors on liver disease progression in chronic hepatitis B carriers in Cambodia.

S gene mutant strains of HBV were isolated from 17 mothers and 2 children. The overall S gene mutation rate of HBV among HBV DNA positive sera was 23.94% in mothers and 18.18% in children, 24.24% in genotype C and 18.75% in genotype B. This rate was lower than that reported from Singapore (39%) [33] but is higher than Thailand (22.4%) [34] and Malaysia (9%) [35]. By this study, high S gene mutation rate of HBV among mother-child pairs of Cambodia suggested the potential spread of vaccine escapes mutant strains in Cambodia. S gene mutation of HBV specifically *a* mutant was occurred most frequently among immunized children and who received plasma derived HepB vaccine [12] and the similar results were found among immunized children of our study but there was no statistically significance. The vaccine itself driven S gene mutation through immune pressure causing amino acid substitution and point mutation [36] although we could not exclude MTCT of S gene mutants.

In our study, only 2 out of 17 children born to mothers with S gene mutants of HBV became infected and both of them did not receive HepB-BD. But, no infection was found if the children received HepB-BD. This could be explained by the hypothesis, that the S gene mutant strain of HBV itself has lower replication rate and also has negative effect on replication of wild type HBV in mixed infection through high T cell immune response causing less infectivity and transmissibility of HBV infection [37]. If the child had received HepB-BD within 24 h, the vaccine totally interrupts MTCT. If the child missed HepB-BD, it causes high possibility of MTCT despite previous study reported on low level of viral replication among mutant strains. Although it was not clear whether S gene mutants of HBV were transmitted vertically or only under immune pressure due to vaccination in our study and the number of isolated mutant strains was quite small to compare, it was revealed that HepB-BD is crucial for preventing MTCT of HBV either wild type or S gene mutants.

S gene mutation of HBV was profoundly occurred in genotype C in our study than genotype B. In fact, genotype B was documented to have high potential for occurrence of amino acid substitution than genotype C [38]. This discrepancy might be due to difference genotype distribution pattern. But the existence of S gene mutants of HBV in Cambodia alarms the possible breakthrough infection among immunized children which may threaten the long term effect of massive immunization. Despite the successful establishment of HepB vaccination, Cambodia has no specific program and protocol for PMTCT of HBV until now. It is challenging for Cambodia on its pathway to meet WHO’s viral hepatitis elimination goal of by 2030. Therefore, the health sector should develop and disseminate the national guideline, HBV screening, assurance of HepB-BD administration to all newborns within 24 h after delivery and provide specific anti-viral treatment to HBV carrier mothers.

Apart from S gene mutation, preS deletion (22.58%), double (48.39%) and combination mutation (32.26%) were also found in HBV genotype C1 strains. In fact, HBV genotype C can easily mutate [39] and its mutation is significantly related to the HCC occurrence [10, 27, 40]. In our study, although we could not correlate the mutant variant with respective liver disease condition, based on recently published study [40], it indicates the need of proper counseling, early and proper referral to the specialized center, assessment for eligibility to anti-viral therapy and regular follow-up care which should be offered to them even they are currently asymptomatic.

This study used the DBS samples to detect not only the HBV sero-markers but also HBV DNA and consequently both partial and full length genome sequences, which is the critical tool for the advanced molecular epidemiology. According to recent systematic review and meta-analysis report, the pooled estimate of sensitivity and specificity for HBV-DNA using DBS was 95% (95% CI: 83–99) and 99% (95% CI: 53–100), respectively [14]. Despite the whole blood samples by venipuncture still ranks as the gold standard for biological specimen, this study proved the capable of DBS for HBV full-length genomes sequences and it is useful as alternative blood collection tool for large scale molecular epidemiological study especially in resources limited countries which may accelerate the surveillance of target virus.

The limitations were present in this study. Firstly, our study could not evaluate the S gene mutation rate by type of HepB vaccine used in the children. Secondly, the study is cross-sectional so that the investigation of liver disease stages and their progress is impossible. Based on the previous study, we could only suggest that HBV C1 infected participants of our study have high possibility to HCC occurrence [40]. At last, even we used DBS samples for detection of partial and full-length HBV genome sequence; we could not compare it with gold standard venous blood samples. Further comparative study on detection of viral genomes in both DBS and venous samples is needed.

## Conclusion

A partial and full-length HBV genomes sequences can be extracted from dried blood spot samples which confer up to molecular epidemiological study of HBV. HBV Genotype C is predominant type in Cambodia but the genotype B is exclusively found in the regions border to Vietnam which shows the historical relation of HBV across the border regions. The recombinant sub-genotype B/C and S gene mutant variants of HBV later known as vaccine escapes mutation (among HBV DNA positive sera, 24.29% in mothers and 16.67% in children, 24.24% in genotype C and 18.75% in genotype B) were found by this study. The double (48.39%) and combination mutation (32.26%) in HBV C1 strains of this study also alarm for the high possibility of hepatocellular carcinoma in individuals with chronic hepatitis B. Therefore, our findings strongly call for implementation of effective countermeasure and its surveillance on viral hepatitis including PMTCT so that Cambodia can continue straightforwardly to meet WHO’s elimination goal of viral hepatitis by 2030.

## Data Availability

The dataset used and analysed during the current study is available from the corresponding author on reasonable request. All the full and partial sequences of HBV isolated in this study are registered to GenBank via DDBJ. The sequences can be accessed at http://getentry.ddbj.nig.ac.jp/top-e.html with accession number of LC522090-LC522130 for HBV partial sequences and LC535909-LC535950 for HBV full sequences. If there is any trouble in accessing the data, the sequences are available from the corresponding author upon reasonable request.

## Abbreviations

HBV: Hepatitis B virus
DBS: Dried Blood Spot samples
HBV DNA: Hepatitis B virus Deoxyribonucleic acid
HIV: Human Immunodeficiency Virus
WHO: World Health Organization
HepB: Hepatitis B vaccine
HBsAg: Hepatitis B surface Antigen
NIP: National Immunization Program
MTCT: Mother-to-child transmission
PMTCT: Prevention of Mother-to-child transmission
HBIG: Hepatitis B Immunoglobulin
CLEA: Chemiluminescent Enzyme Immunoassay
PCR: Polymerase chain reaction
HepB-BD: Hepatitis B vaccination birth-dose
